# A patient full of surprises: a body packer with cocaine intoxication, pneumococcal pneumonia and HIV infection

**DOI:** 10.1186/s12873-018-0180-7

**Published:** 2018-09-04

**Authors:** Miriam Luginbühl, Timo Junker, Dagmar I. Keller

**Affiliations:** 1 Department of Internal Medicine, University Hospital Zurich, Rämistrasse 100, 8091 Zurich, Switzerland; 2 Emergency Department, University Hospital Zurich, Rämistrasse 100, 8091 Zurich, Switzerland

**Keywords:** Body packer, Cocaine, Sepsis, Human immunodeficiency virus, Pneumococcal pneumonia

## Abstract

**Background:**

Smuggling of illegal drugs by hiding them inside one’s own body, also called body packing, is a worldwide phenomenon. Cocaine is the most frequently transported drug. Body packing is a potentially lethal practice. The most serious complications of body packing are gastrointestinal obstruction or perforation and drug toxicity due to packet leakage or rupture.

**Case presentation:**

A 30-year-old confirmed body packer was brought to our emergency department from jail because of agitation and mydriasis. He presented with a high respiratory rate of 40/min but normal oxygen saturation on ambient air, a heart rate of 116 bpm, a blood pressure of 116/68 mmHg and a temperature of 38.0° Celsius. Blood tests were suggestive of infection, urine analysis was positive for cocaine. Abdominal and thoracic computed tomography scans showed pulmonary infiltrates as a possible focus of infection; signs of bowel obstruction or perforation were absent. Given his clinical presentation, we suspected severe infection rather than massive cocaine intoxication to be the main problem. We therefore withheld immediate surgical decontamination. Instead, we started broad-spectrum antibiotic treatment with piperacillin/tazobactam plus clarithromycin for suspected severe community-acquired pneumonia or abdominal sepsis and treated the patient with intravenous midazolam for symptomatic cocaine intoxication. After detection of urinary pneumococcal antigen, the antibacterial regimen was changed to ceftriaxone and vancomycin for pneumococcal pneumonia. In addition, we found human immunodeficiency virus (HIV) type 1 infection as underlying disease. The patient recovered from his acute illness and was discharged after 7 days of treatment with ceftriaxone plus vancomycin. Antiretroviral therapy was started in an outpatient setting.

**Conclusions:**

With this case report, we emphasize the need to look for alternative diagnoses to intoxication and gastrointestinal obstruction in acutely ill body packers with atypical presentation. Special risks, such as underlying HIV infection and potential antimicrobial resistance according to the individual’s geographical origin, should be taken into account while treating these patients.

## Background

Smuggling of illegal drugs by hiding them inside one’s own body is a worldwide practice. Persons who swallow or insert drug-containing packets into a body cavity are called “body packers”. Cocaine is the most frequently transported drug, followed by heroin, metamphetamine, opium, marijuana and hashish [1–4]. According to the literature, a single body packer usually carries 50–100 packets [1]. A recent Swiss single center study showed an average number of 34 packets per body packer, with numbers ranging from 1 to 100 [3]. Each packet usually contains 8-10 g of drug, which is a potentially lethal dose in case of cocaine, amphetamines and heroin [1]. Different types of drug packages have been described: Loosely packed cocaine powder in wrappings such as condoms is referred to as “type 1” package. Tightly packed cocaine powder or paste in multiple tubular latex layers is referred to as “type 2” or “type 3” package, the latter having an additional covering of foil [4]. The most recently described type of package, “type 4”, consists of a hardened cocaine paste wrapped in tubular latex covered with paraffin or fiberglass [4, 5]. These improvements in the quality of the drug packages have resulted in a minimized risk of leakage or rupture. Hence, nowadays most body packers remain asymptomatic and can be managed conservatively with osmotic laxatives and/or polyethylene glycol (PEG), and possibly gut motility increasing agents [1, 3, 6].

Possible complications of body packing are gastrointestinal obstruction or perforation and drug toxicity, called “body packer syndrome” [1, 5]. According to case series, gastrointestinal obstruction occurs in about 5% of body packers with a stable incidence over time [5]. Management of these patients is surgical. In the earlier years of experience with body packer syndrome, removal of the drug packages through multiple enterotomies in an unprepared intestine was advocated to avoid excessive handling of the packets, which was considered dangerous because of their fragility [5]. Due to the improved drug packaging methods, a more conservative surgical approach was recommended. With or even without enterotomy packets can be “milked” along the intestine until impaction is resolved [1, 5].

Drug toxicity due to package leakage or rupture is reported less frequently than gastrointestinal obstruction, probably because of increasing quality of drug packages over time [3, 6, 7]. In case of cocaine-containing packets, rupture is life-threatening and immediate surgical evacuation of the drug containers is the recommended treatment [8]. Additionally – during preparation for surgery – symptomatic treatment of cocaine intoxication should be started including benzodiazepines for agitation or seizures, calcium channel blockers or nitrates for hypertension, sodium bicarbonate to prevent ventricular arrhythmia in case of a broad QRS complex, and physical cooling for hyperthermia [1, 9].

In addition to body packer syndrome, there are other potentially challenging aspects in the care of body packers. Communication between patient and medical staff can be difficult due to language barriers and sociocultural differences. This complicates medical history-taking and informed consent for invasive examinations, radiological studies or therapeutic interventions. Also, potential underlying diseases according to the patient’s geographical and social origin should be taken into consideration and investigated if clinically appropriate.

We present a case of an agitated and unwell cocaine body packer brought to our Emergency Department (ED) from jail, in whom we found severe pneumonia and HIV infection as the main reasons for his deteriorating state of health in addition to cocaine intoxication. This case is reported according to the CARE checklist [10].

## Case presentation

**Fig. 1:**
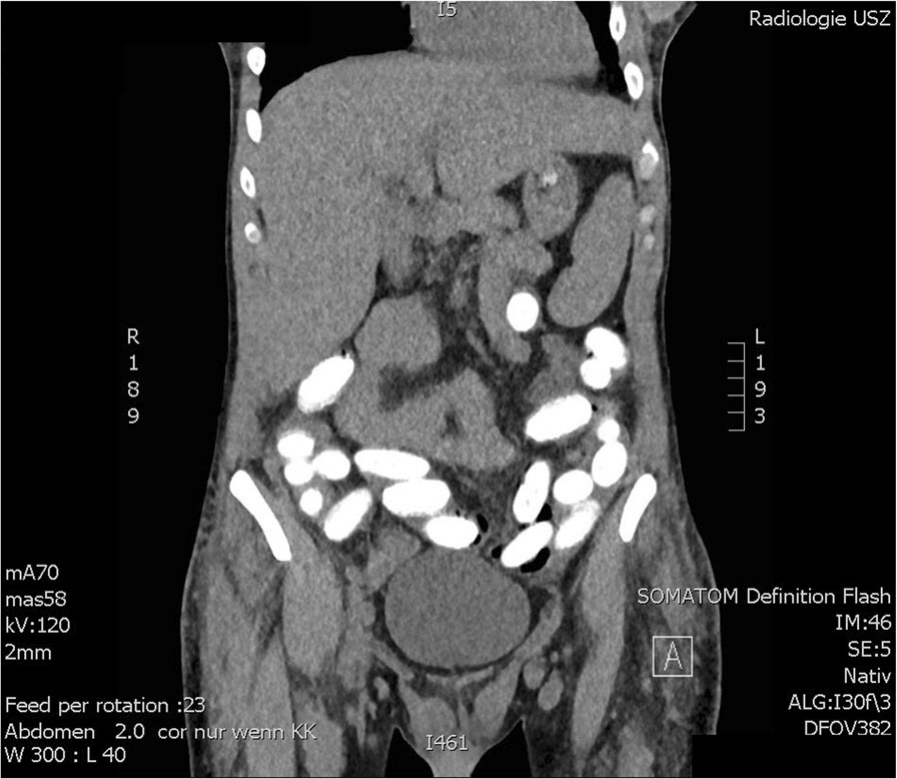
Computed tomography scans at the initial presentation showing multiple drug packages in the patient’s gastrointestinal tract

A 30-year-old Brazilian male was brought to our ED from a local jail because of agitation. He had been arrested by Swiss authorities because of suspected internal concealment of drugs of abuse (body packing), which had been confirmed by abdominal CT scan in our hospital approximately 12 h before (Fig. 1). During this first visit, the patient had presented asymptomatic and had admitted to carrying cocaine-containing body packets. In addition, he had reported recreational use of marijuana and cocaine. It was also noted that the patient was transsexual; he had breast implants. The patient’s past medical history was unknown. Standard operating procedure in such a case is to perform a CT scan without a consultation at the ED. During the second visit approximately 12 h later, the patient presented with psychomotor agitation, mydriasis and tachycardia. His heart rate was 116 bpm, blood pressure was 116/68 mmHg, respiratory rate was 40/min, oxygen saturation by pulse oximetry was 99% on ambient air and auricular temperature was 38.0° Celsius. Communication with the patient was impossible because of his altered mental status and because he spoke a foreign language. The physical examination of heart, lungs and abdomen revealed no pathologies. Neurological examination showed symmetrical spontaneous movement of all extremities and symmetrical gaze to both sides prompted by speech or touch. Glasgow coma scale was 11. An electrocardiogram showed sinus tachycardia without signs of ischemia. Due to our knowledge of the ingested body packets, we first suspected cocaine intoxication because of package rupture. We treated the patient with repeated doses of intravenous midazolam and performed an emergency abdominal CT scan to guide potential emergency surgical decontamination. The CT scan showed 60–70 packets in the gastrointestinal tract without signs of gastrointestinal obstruction or perforation. Laboratory results showed an increased C-reactive protein (CRP) level (231 mg/l, norm < 5 mg/l), an increased creatinine level (181 mcmol/l, norm 62–106 mcmol/l), an increased creatine kinase (CK) level (1000 U/l, norm < 190 U/l), a slightly increased troponin level (16 ng/l, norm < 14 ng/l), hypoglycemia (2.8 mmol/l), mild hyponatremia (128 mmol/l, norm 136–145 mmol/l), hyperkalemia (5.8 mmol/l, norm 3.3–4.5 mmol/l) and hyperphosphatemia (1.68 mmol/l, norm 0.87–1.45 mmol/l) (Table 1). Urine analysis showed no evidence of urinary tract infection. A qualitative urine toxicological test was positive for cocaine and benzodiazepines.

**Table 1:** Blood tests

	value at admission	normal range
Sodium	128	136–145 mmol/l
Potassium	5.8	3.3–4.5 mmol/l
Calcium total	2.07	2.09–2.54 mmol/l
Calcium corr.	2.35	2.09–2.54 mmol/l
Phosphate	1.68	0.87–1.45 mmol/l
Glucose (fasting)	2.8	3.4–5.6 mmol/l
Creatinine	181	62–106 mcmol/l
Urea	22.7	2.14–7.14 mmol/l
Lactate dehydrogenase	579	240–480 U/l
Aspartate aminotransferase	81	< 50 U/L
Alanine aminotransferase	18	< 50 U/L
Gamma-glutamyltransferase	11	< 60 U/L
Alkaline phosphatase	61	40–130 U/l
Bilirubin	9	< 21 mcmol/l
Albumin	29	40–49 g/l
C reactive protein	231	< 5 mg/l
Creatine kinase	1000	< 190 U/l
Troponin	16	< 14 ng/l
Cortisol	686	74–286 mmol/l
Hemoglobin	75	134–170 g/l
Hematocrit	0.233	0.4–0.5
Erythrocyte count	2.76	4.2–5.7 × 10^12^/l
Mean corpuscular volume	84.4	80–100 fl
Mean corpuscular hemoglobin	27.2	26–34 pg
Reticulocyte count	81	27–132 10^9^/l
Platelet count	161	143–400 10^9^/l
White blood cell count	7.92	3.0–9.6 10^9^/l
Neutrophil count	4.36	1.4–8.0 10^9^/l
Monocyte count	1.43	0.16–0.95 10^9^/l
Lymphocyte count	2.12	1.5–4.0 10^9^/l
INR	1.4	< 1.2

**Fig. 2:**
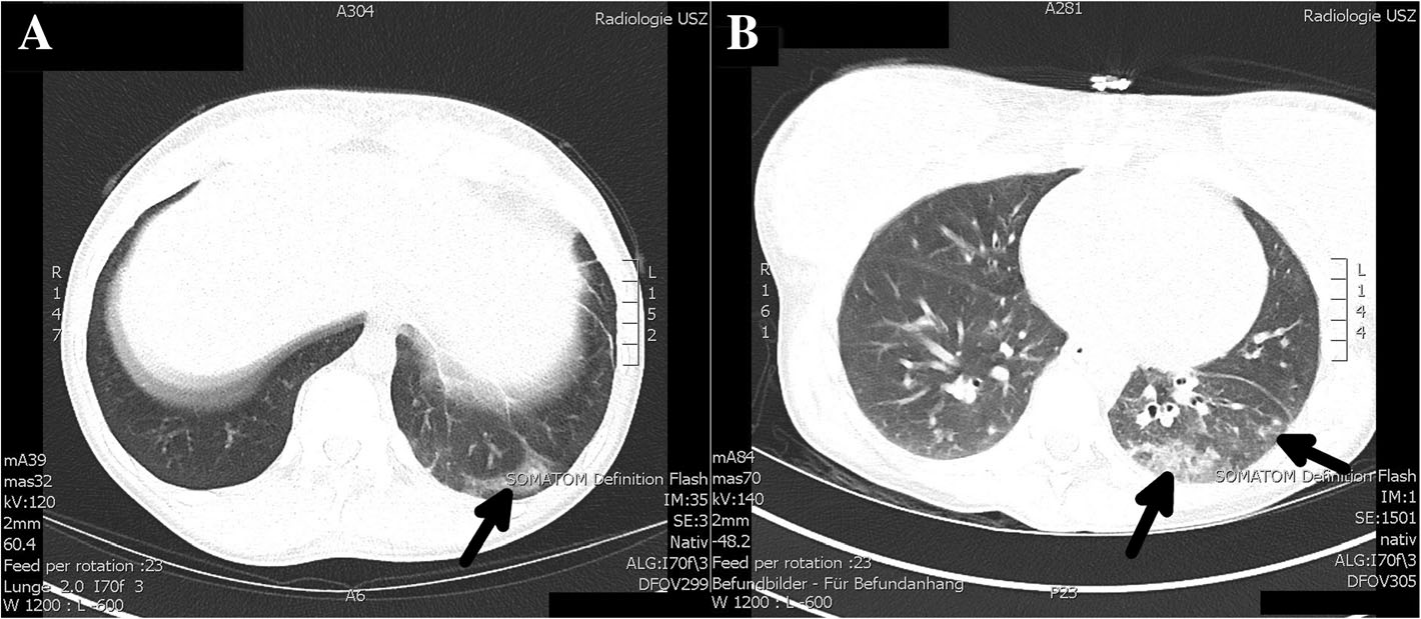
Computed tomography scans at the initial presentation (**a**) and 14 h later (**b**) showing progressive pulmonary infiltrates (arrows)

Given the clinical presentation of our patient with remarkable hypotension despite cocaine intoxication and elevated inflammatory markers, we suspected infection rather than massive cocaine intoxication to be the main problem. Thorough review of the abdominal CT scans revealed rapidly progressive pulmonary infiltrates in the lower left lobe and lingula, diffuse lymphadenopathy and hepatosplenomegaly (Fig. 2). To assess the extent and morphology of the pulmonary infiltrates and to investigate for thoracic lymphadenopathy or solid tumors, we performed a thoracic CT scan. Compared to the abdominal CT scan 4 h before, it showed progression of the pulmonary infiltrates in the lower left lobe and lingula, multiple nodular pulmonary consolidations in both upper lobes compatible with septic emboli or tuberculous foci as well as diffuse cervical, mediastinal and axillary lymphadenopathy.

Due to the above named findings, we withheld emergency laparotomy and started broad-spectrum antibacterial treatment with ceftriaxone and clarithromycin according to our hospital’s guidelines for severe pneumonia. Given the rapid progression of the pulmonary infiltrates in the course of few hours on the thoracic CT scan, we considered the possibility of toxic rather than infectious infiltrates. Because of the patient’s poor general condition we therefore decided to broaden the antibacterial treatment as for sepsis with unknown focus – especially taking into account an abdominal source – to piperacillin/tazobactam instead of ceftriaxone according to our hospital’s guidelines. Based on the elevated creatinine level we diagnosed acute kidney injury, most probably caused by dehydration precipitated by infection and cocaine intoxication. The patient also had an elevated CK level. Cocaine-induced rhabdomyolysis could therefore be an additional cause of the acute kidney injury. Therefore we treated the patient with intravenous fluids.

The patient was admitted to our intensive care unit and isolated for possible pulmonary tuberculosis. In the course of his hospitalization, further diagnostics were available: Pneumococcal urinary antigen test was positive. Screening and confirmatory test for HIV-1 were positive and CD4+ T-cell count was 144/μl, so we diagnosed CDC stage A3 HIV infection. Blood and urine cultures didn’t show any bacterial growth. The diagnosis of pneumococcal pneumonia and sepsis could thus be confirmed, with underlying untreated HIV infection as risk factor. Active pulmonary tuberculosis was excluded by three negative sputum smears and three negative sputum cultures.

With the aid of an interpreter, the patient told us in the course of the hospitalization that one of the drug packages had ruptured in his mouth during swallowing, but that he had been able to spit out most of the cocaine. The exact time of this event remained uncertain.

Upon diagnosis of pneumococcal pneumonia, the antibacterial therapy was changed from piperacillin/tazobactam back to ceftriaxone. As beta-lactam resistant pneumococci were a concern because of the patient’s geographical origin, vancomycin was added to ceftriaxone. This regimen was continued for 7 days. Clarithromycin was stopped after diagnosis of pneumococcal pneumonia. After 9 days, the patient was discharged into custody of the Swiss authorities. Antiretroviral therapy was established in an outpatient setting after ruling out active pulmonary tuberculosis. The patient recovered uneventfully.

**Fig. 3:**
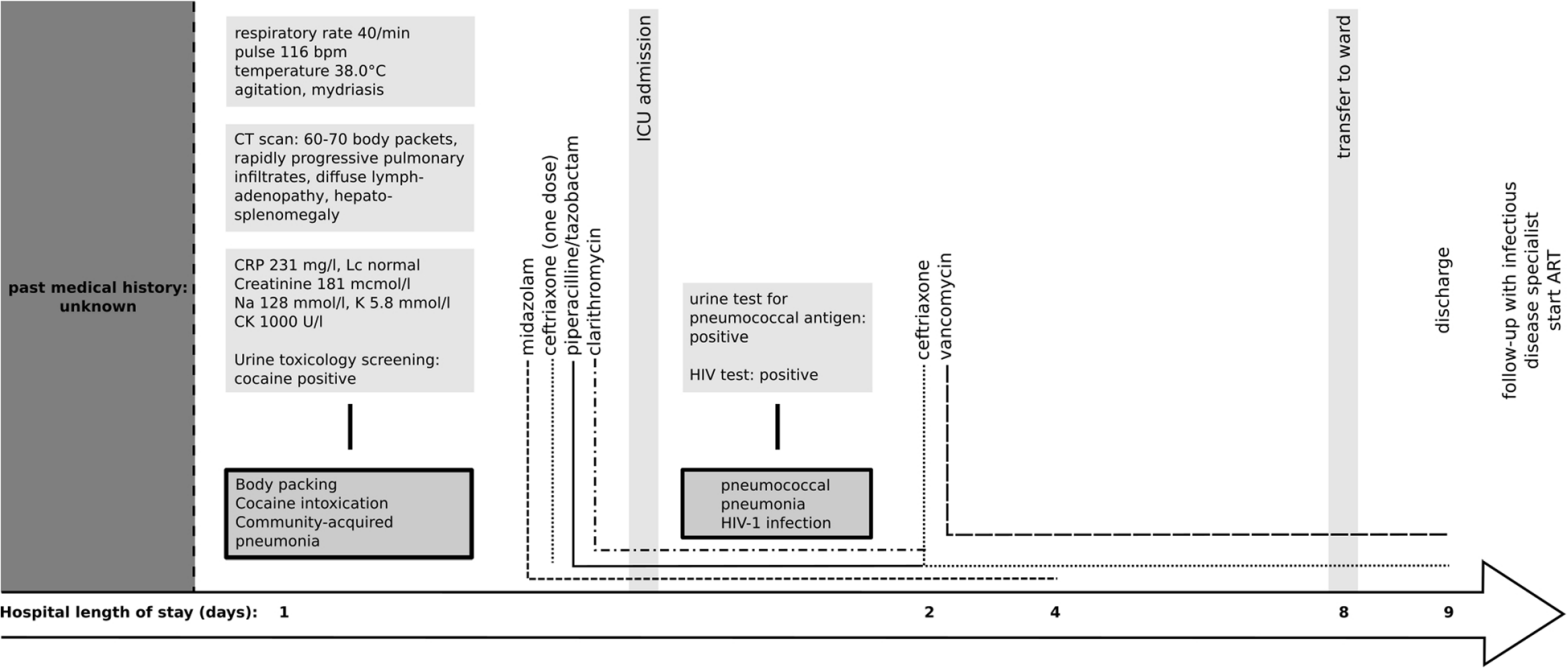
**Timeline.** Abbreviations: min = minutes; bpm = beats per minute; C = Celsius; CT = Computed Tomography; CRP = C-Reactive Protein; Lc = leukocytes; Na = sodium; K = potassium; CK = creatine kinase; ICU = Intensive Care Unit; HIV = Human Immunodeficiency Virus; ART = Antiretroviral Therapy

Figure 3 shows the key diagnostic findings and interventions on a timeline.

## Discussion

Our case describes a cocaine body packer with cocaine intoxication, pneumococcal pneumonia with sepsis and HIV infection as underlying disease.

The rupture of one drug package during swallowing explains the detection of cocaine in the patient’s urine with our qualitative assay, which is an immunological test for benzoylecgonine. Positive urine test for a cocaine metabolite even without package rupture has also been described and attributed to residues of cocaine on the surface of the packages [8]. In our patient we found signs of cocaine intoxication, which we considered as mild compared to what we would expect after rupture of an internal drug package. Given this and the good clinical response to treatment with midazolam, we decided against emergency surgery and continued conservative management with cardiopulmonary monitoring and sedatives. Successful conservative management of mild cocaine intoxication in body packers has also been described in literature [11].

In our patient, we diagnosed pneumococcal pneumonia and HIV infection as cause for his deteriorating state of health. To our knowledge, significant co-morbidities are rarely reported in body packers [11, 12]. This could represent the true population characteristics of body packers, but it could also be due to difficulties in medical history-taking (language barriers, bad general condition of the patients or deliberate concealment by the patients). However, as body packing is a dangerous venture without major financial profit for the individual body packer, we expect individuals of mostly low socio-economic status to act like this. These persons might also be involved in other potentially health-risky behavior, such as prostitution or drug abuse. In case of clinical suspicion, underlying diseases such as HIV infection and opportunistic pathogens should be considered in the differential diagnosis. Specific antimicrobial resistance according to the individual’s geographic origin is another challenge to meet, especially in the treatment of critically ill patients.

## Conclusions

We emphasize the importance of looking for alternatives to intoxication and gastrointestinal obstruction as cause of acute illness in body packers with atypical presentation. Special risks, such as underlying HIV infection and antimicrobial resistance according to the individual’s geographical origin should be taken into account when treating these patients.

## Abbreviations

CDC: Centers for disease control and prevention
CK: creatinine kinase
CRP: C-reactive protein
CT: computed tomography
ED: Emergency department
HIV: human immunodeficiency virus
PEG: polyethylene glycol
